# A novel *CTBP1* variant in a Chinese pediatric patient with a phenotype distinct from hypotonia, ataxia, developmental delay, and tooth enamel defect syndrome

**DOI:** 10.3389/fgene.2024.1344682

**Published:** 2024-01-29

**Authors:** Qiang Zhang, Yusi Liu, Xuan Liu, Yue Zhao, Jihong Zhang

**Affiliations:** ^1^ Hematology Laboratory, Sheng Jing Hospital of China Medical University, Shenyang, China; ^2^ The Maternal and Child Health Care Hospital of Guangxi Zhuang Autonomous Region, Guangxi Birth Defects Prevention and Control Institute, Nanning, China

**Keywords:** C-terminal binding protein 1 (CTBP1), HADDTS, *de novo* variant, whole-exome sequencing, clinical heterogeneity

## Abstract

Hypotonia, Ataxia, Developmental Delay, and Tooth Enamel Defect Syndrome (HADDTS) is an exceptionally rare disorder resulting from a heterozygous variant in the C-terminal binding protein 1 (*CTBP1*) gene. To date, a mere two variants (14 patients) have been documented on a global scale. The aim of this study was to identify a causative *CTBP1* variant in a Chinese patient, and to determine the potential pathogenicity of the identified variant. Here, Whole-exome sequencing (WES) was conducted on the proband to pinpoint the candidate variant. Following this, Sanger sequencing was employed to validate the identified candidate variant and examine its co-segregation within the available family members. Employing both *in silico* prediction and three-dimensional protein modeling, we conducted an analysis to assess the potential functional implications of the variant on the encoded protein. Our investigation led to the identification of a novel heterozygous variant in the *CTBP1* gene, namely, c.371 C>T (p.Ser124Phe), in a Chinese patient. This case represents the first confirmed instance of such a variant in a Chinese patient. When comparing the patient’s clinical symptoms with those reported in the literature, notable distinctions were observed between her primary symptoms and those associated with HADDTS. She showed other signs such as microcephaly, coarse facial features, single transverse palmar crease, visible beard, myopia, coarse toenail and skeletal anomalies. This study enriching the spectrum of genetic variants observed in different ethnic populations and expanding the phenotypic profile associated with this gene. These findings are expected to contribute to the enhancement of future variant-based screening and genetic diagnosis, while also providing further insights into the pathogenic mechanisms underlying *CTBP1*-related conditions.

## Introduction

C-terminal binding protein 1 (CTBP1), located on chromosome 4p16, functions as a transcriptional co-repressor (Chen, 2021). Additionally, CTBP1 serves as a crucial scaffolding protein that recruits chromatin-modifying enzymes to specific genomic regions, thereby coordinating gene expression programs (Sekiya et al., 2021). *CTBP1* plays a regulatory role in gene expression during various processes, including development, apoptosis, and oncogenesis (He et al., 2021). Initially, CTBP1 was discovered as a protein capable of binding to the C-terminus of the human adenovirus E1A protein (Hildebrand and Soriano, 2002; Senyuk et al., 2005). Studies in mice have demonstrated that *CTBP1* has specific transcriptional functions during fetal development (Stankiewicz et al., 2014; Blevins et al., 2017). Furthermore, Hu et al. (2019) uncovered the protective effect of *CTBP1* on hippocampal and cortical neurons, safeguarding them against degeneration. Notably, high *CTBP1* expression has been shown to attenuate apoptosis and enhance neuronal activity in these specific neuronal populations (Stankiewicz et al., 2014). In 2016, it was definitively established that variant in the *CTBP1* gene, located at chr4:1,205,233–1,242,918 GRCh37/hg19, cause Hypotonia, Ataxia, Developmental Delay, and Dental Enamel Defect Syndrome (HADDTS, MIM:617915) (Beck et al., 2016). In this study, we present a unique phenotype resulting from a novel *de novo* variant in the *CTBP1* gene, which represents the first confirmed case in a Chinese patient.

## Materials and methods

### Next-generation sequencing

The patient underwent a whole-exome sequencing examination, wherein a genomic DNA sample was collected and utilized to construct sequence libraries. The construction of libraries was carried out using the Agilent Sure Select Human Whole Exome V2 Kit (Agilent Technologies, Santa Clara, CA). Subsequently, sequencing of the prepared libraries was performed using the HiSeq2500 System (Illumina, San Diego, CA). The reads obtained from the Burrows-Wheeler Aligner (BWA) software package (v. 0.7.15) were aligned to the human reference genome (GRCh37/hg19). Variant calling and annotation utilized the Genome Analysis Toolkit (GATK), and further variant annotation and prioritization were conducted using TGex software (LifeMap Sciences, Inc. v5.7).

### Sanger sequencing confirmation

Sanger sequencing was conducted to validate the variant in the proband and her family members. The designed primers, 5′-CTG​CCA​CGT​GAA​CCT​AAA​GT-3′ and 5′-GTG​TGT​TAT​CTG​CAT​GTG​CC-3′, obtained from Oligo7, were utilized specifically for c.371 C>T (p.Ser124Phe).

### Bioinformatic analysis and verification of observations

The bioinformatics tools Scale Invariant Feature Transform (SIFT, http://sift.jcvi.org/), Mutation Taster software (https://www.mutationtaster.org/), Provean (http://provean.jcvi.org/seq_submit.php), Combined Annotation Dependent Depletion (CADD, https://cadd.gs.washington.edu/snv) and rare exome variant ensemble learner (REVEL,https://sites.google.com/site/revelgenomics/) were applied to predict the impact of the variant on protein function. The protein 3D structures of CTBP1 were generated by the Swiss-Model server (https://swissmodel.expasy.org/), and the ACMG/AMP variant classification guidelines were employed for variant classification (Li and Wang, 2017).

## Results

### Clinical data

The Chinese patient arrived at our hospital exhibiting global developmental delay, dwarfism, and distinctive facial features. She was a full-term baby born to non-consanguineous parents, yet she was small for her gestational age (SGA). No signs of neonatal asphyxia were present during delivery, and she achieved perfect Apgar scores of 10/10/10. During the physical examination, we noted her birth data: a height of 46 cm (−2 SD), weight of 2.4 kg (−2 SD), and head circumference of 33 cm (−1.5 SD).

**FIGURE 1:**
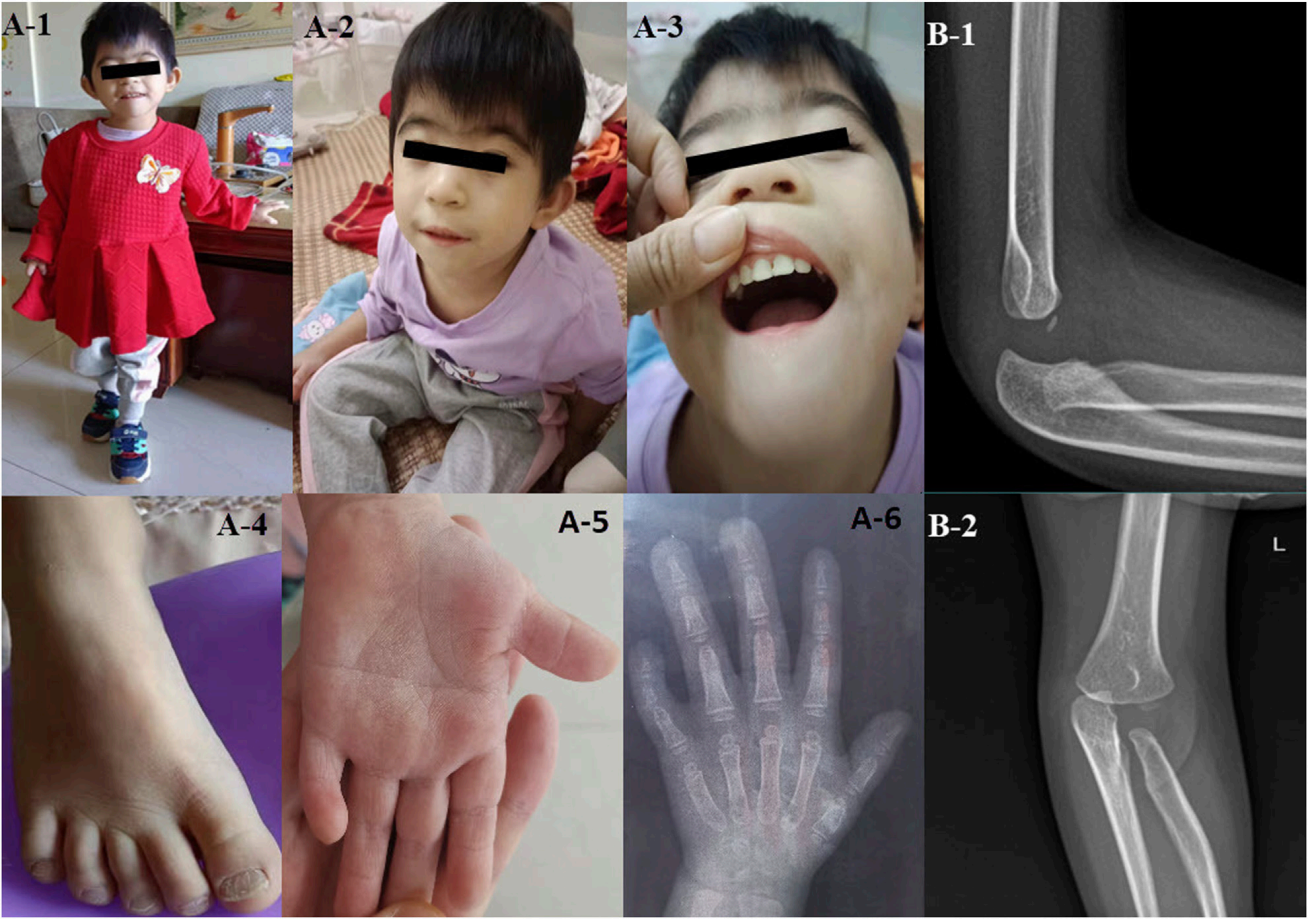
The clinical features of the proband with *CTBP1* variation **(A-1–A-6)** illustrate the phenotypic characteristics of the patient: microcephaly, a visible beard, synophrys, low-set ears, protruding ears, coarse toenail, a single transverse palmar crease, and a short fifth finger. **(B-1, B-2)** depict the congenitally dislocated radial head of the patient.

The child displayed unique facial attributes such as microcephaly, a visible beard, synophrys, low-set and protruding ears. She also had other physical characteristics, including a single transverse palmar crease, a short fifth finger, congenital dislocation of the radial head, pectus excavatum, and myopia (Figure 1).

**FIGURE 2:**
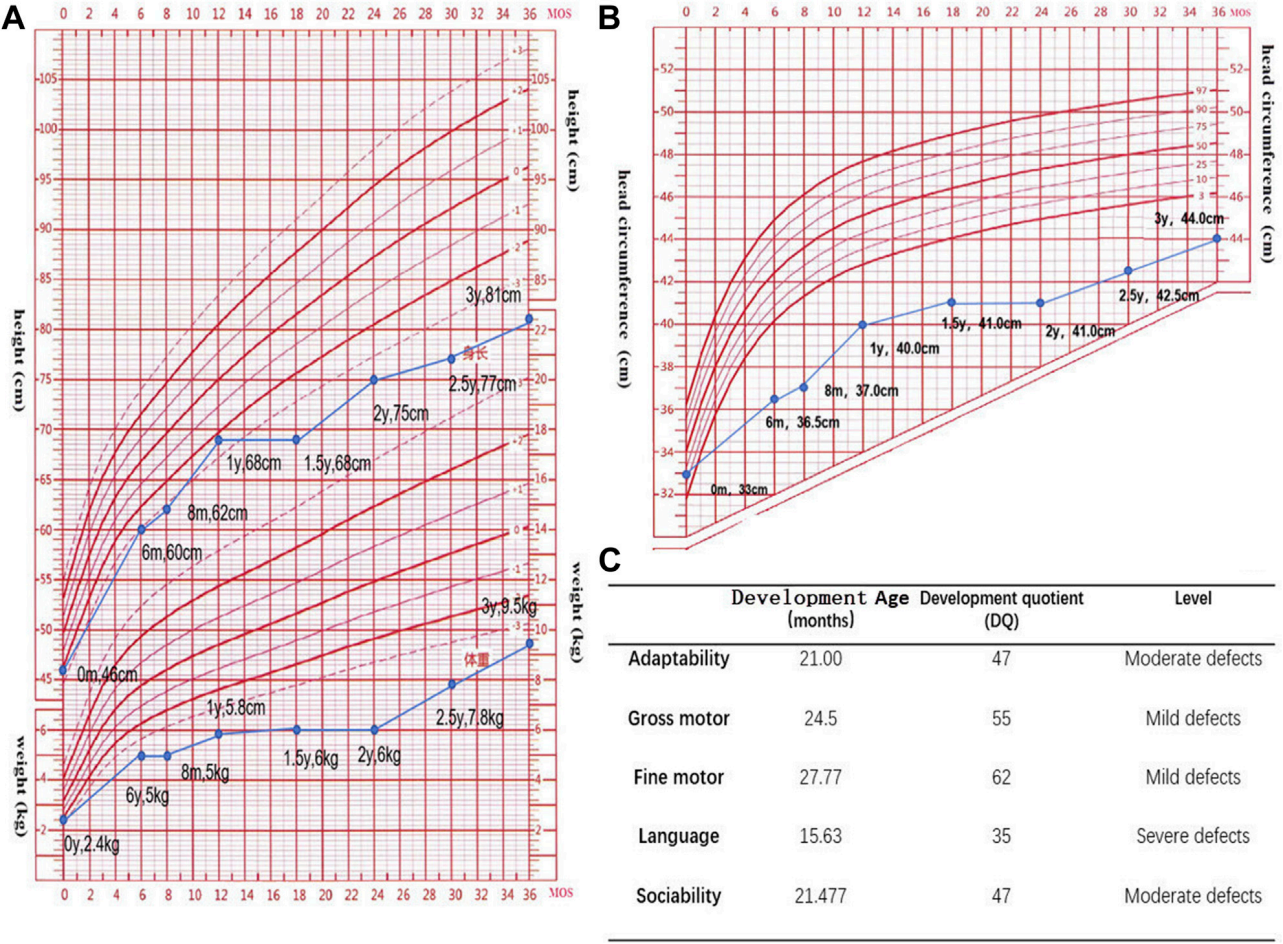
**(A,B)**. Standard deviation scores (SDS) for height, weight, and head circumference in the age range of 0–3 years. (The data suggests that the child is experiencing growth and developmental delays, as evidenced by parameters such as height, weight, and head circumference falling below the averages for their age group.) **(C)**. The Gesell Developmental Scale (GDS) has been used to assess neurodevelopment in the patient. The GDS reveals slight shortcomings in motor development, moderate inadequacies in adaptive and social skills, and profound impairments in language development.

Assessments using the Gesell Developmental Scale (GDS) (Figure 2C) and the Infant-Junior High School Student’s Social Living Ability Scale (S-M) (with the patient’s score exceeding 8 points) indicated that the child was developmentally delayed and had difficulties adjusting socially. Her intelligence quotient (IQ) score of 49 suggests a moderate level of intellectual disability, as per the Wechsler Preschool and Primary Scale of Intelligence (WPPSI) scale, where moderate intellectual impairment falls within the range of 35–49.

Additionally, a cardiac ultrasound revealed mild tricuspid valve regurgitation. However, tests for liver function, kidney function, 25-OH-VD levels, thyroid function, female endocrine function, adrenal gland function, electroencephalogram, blood tandem mass spectrometry, and brain magnetic resonance imaging (MRI) all returned normal results.

### Genetic testing

**FIGURE 3:**
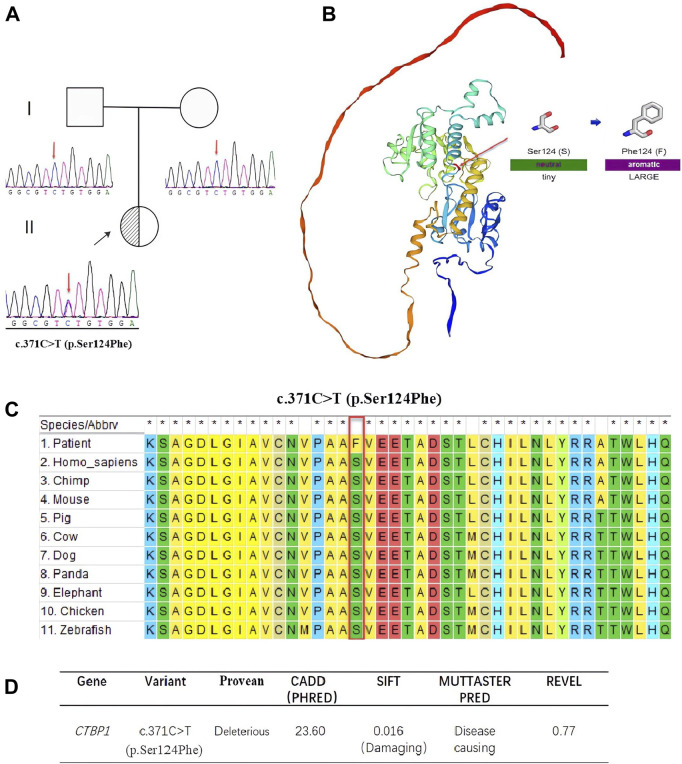
**(A)**. Sequencing results of c.371 C>T (p.Ser124Phe) and pedigree chart of the patients’ family. Sanger sequencing DNA chromatograms of *CTBP1* indicate that the variant is *de novo*. The proband is marked with a black arrow. **(B)**. Three-dimensional structures of CTBP1 protein. The residue at sequence position 124 in this protein is serine, which has a neutral side chain. The variant residue is phenylalanine, which has an aromatic side chain. **(C)**. Sequence alignment shows that Ser124Phe is highly conserved across various species. **(D)**. The effects of c.371 C>T (p.Ser124Phe) were predicted using five different software packages.

We identified a heterozygote variant, c.371 C>T (p.Ser124Phe), in the *CTBP1* gene (NM_001328.2, Chr4:1,219,324 in exon 4) in the proband using WES. Sanger sequencing confirmed that the c.371 C>T (p.Ser124Phe) variant was a *de novo* variant (see Figure 3A). We used five *in silico* tools to predict the impact of the novel variant (see Figure 3D), which suggested that c.371 C>T (p.Ser124Phe) is a harmful variant. The three-dimensional structure of the variant protein and the pathogenicity of amino acid substitutions and their molecular mechanisms were analyzed using the SWISS-MODEL software and MutPred2 web (Pejaver et al., 2020), respectively (see Figure 3B). It was observed that the variant protein altered ordered interfaces, transmembrane proteins, metal binding, and resulted in the loss of the catalytic site at position N119 and the acquisition of a heterologous site at position P121.

## Discussion

HADDTS is an extremely rare autosomal dominant disorder caused by a disruptive pathogenic variant of *CTBP1*. Fourteen cases of pathogenic variants in the *CTBP1* gene have been reported globally, in such cases hypotonia, ataxia, developmental delay and dental enamel defects have long been recognized as the core symptoms. Here, we first described a Chinese pedigree with *CTBP1* variant, and interestingly the patient developed none of hypotonia, ataxia, and dental enamel defects.

**FIGURE 4:**
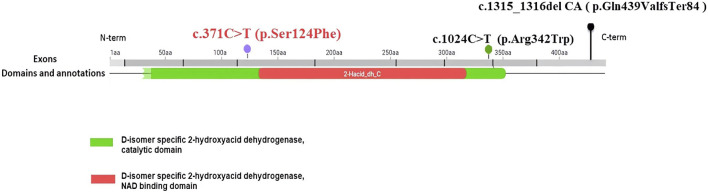
The CTBP1 variants that were reported. c.371 C>T (p.Ser124Phe) in exon 4 and D-isomer specific2-hydroxyacid dehydrogenase, catalytic domain.

The *CTBP1* gene is the only known cause of HADDTS (Bhatia et al., 2020). The gene is widely expressed in brains, livers, lungs, and it may play a key role in controlling the equilibrium between tubular and stacked structures in the Golgi complex, and functioning in brown adipose tissue (BAT) differentiation (Acosta-Baena et al., 2022). The true prevalence of HADDTS is not well-known because only two pathogenic variants (14 patients) in *CTBP1* have been found around the world (Jafari Khamirani et al., 2021). A novel variant is described in this study, c.371 C>T (p.Ser124Phe). The variant was *de novo* and the relatedness was confirmed by paternity testing (PS2). It is a rare Single Nucleotide Polymorphism (SNP), neither found to be present in controls, the 1,000 Genome Project (https://www.internationalgenome.org/) nor the Exome Sequencing Project (https://exome.gs.washington.edu/) (PM2-supporting). Rare SNPs have very broad and deleterious effects on phenotypes when compared with the weak effects of common SNPs (Bomba et al., 2017). Benign missense variant in *CTBP1* has a low rate meanwhile missense variants are common mechanisms of disease (Z-score:3.31 > 3.09, PP2), and multiple pathogenicity prediction algorithms support deleterious effects on genes or gene products (Figures 3C, D, PP3). Thus, the novel variant can be classified as likely pathogenic (PS2, PM2-supporting, PP2, PP3) according to ACMG. It is important to note that, despite the ACMG guidelines assessing this variant as potentially pathogenic, further functional studies are necessary to validate its pathogenicity given the significant deviation in phenotype observed in this patient compared to prior reports. In addition, we summarized all the reported mutants that leads to HADDTS (Figure 4). The clinical presentations of HADDTS include intellectual disability, hypotonia, ataxia, developmental delay, failure to thrive, and tooth enamel defects (Vijayalingam et al., 2020). The patient in this study exhibited different phenotype in comparison with the previously reported cases. She had no signs of hypotonia, difficulty in walking or tooth enamel defect. Meanwhile, the child exhibited distinct facial features including microcephaly, a small beard, synophrys, low-set ears, and protruding ears, as well as other physical characteristics such as prominent palms, a short fifth finger, an inability to fully extend the elbow joint, pectus excavatum, and myopia. We compiled the clinical phenotypes, laboratory test results and molecular characteristics of 14 previously reported cases (Table 1). Through extensive literature analysis, we found that the youngest patient among all the enrolled individuals was 3 years old (our study), while the oldest was 26 years old (Table 1-P14). The male-to-female ratio was 2:1. Growth retardation was observed in 93.3% (14/15) of the patients, and developmental regression was present in 40.0% (6/15), with motor and language regression being the most common. Additionally, 84.6% (11/13) had dental defects, 66.7% (10/15) exhibited ataxia, 93.3% (14/15) developed intellectual disability, and 86.7% (13/15) had decreased muscle tone. All patients showed delayed language development (15/15). The genetic variants identified in this study were primarily attributed to *de novo* variants (14/15), with only one reported case of somatic mosaicism from maternal inheritance (Table 1-P1). Among the 15 patients, 13cases had the same variants (c.991 C > T (p.Arg331Trp) in NM_001012614.1; c.1024 C>T (p.Arg342Trp) in NM_001328.2). These variants were responsible for a high degree of symptom consistency, characterized primarily by developmental delay, intellectual disability, failure to thrive, hypotonia, ataxia, and tooth enamel defects. c.1024 C>T (p.Arg342Trp) is within the C-terminal portion of the PLDLS (Pro Leu-Asp-Leu-Ser) binding cleft, which is the domain through which CTBP1, interacts with chromatin-modifying enzymes and mediates chromatin-dependent gene repression pathways (Quinlan et al., 2006; Beck et al., 2019). The variant discovered in the study, c.371 C>T (p.Ser124Phe), is located in the catalytic domain. Ser124 is a catalytic residue, hence crucial for the protein’s function. The residue at sequence position 124 in this protein is a serine which has a neutral side chain. The variant residue is phenylalanine which has an aromatic side chain (i.e., containing an aromatic ring), that can stack against others. A change from a Ser to a Phe side chain is a very large one, and might well result in a change to the protein’s function. A Ser to Phe residue change is highly unfavoured in terms of conserved amino acid properties (Figure 3C). The newly reported patient is phenotypically different in comparison with the previously reported cases. She had no sign of hypotonia, gait abnormality and dental defect. Initially, we considered the possibility of Cornelia de Lange syndrome of the patient based on her physical features (Sarogni et al., 2020). However, after conducting whole exome sequencing analysis, no genetic variants associated with Cornelia de Lange syndrome, such as *NIPBL, SMC1A, SMC3, RAD21 and HDAC8* were found (Helgeson et al., 2018). As a result, this disease was ultimately ruled out. The patient was small for gestational age infant, which suggested the possibility of intrauterine growth retardation. We closely monitored her growth from birth until the age of 3 (Figure 2) and found that the patient’s growth failure remained unimproved throughout. The clinical presentation of the proband in this study was obviously different from the core clinical symptoms (tooth enamel defects, intellectual disability, and dysarthria) of the previously reported. Although *CTBP1* variants can lead to mitochondrial complex I and IV activities impairments in skeletal muscle, causing mitochondrial diseases (Sommerville et al., 2017), the patient we described did not exhibit any biochemical changes or muscle abnormalities. The exact pathogenic mechanism of the novel discovered heterozygous variant (c.371 C>T (p.Ser124Phe)) has not been fully elucidated. Further observation is required. It remains to be determined whether this patient will gradually manifest phenotypes of both mitochondrial disease and HADDTS as she ages or if this variant in *CTBP1* will result in another disease or subtype alternatively, these symptoms may represent heterogeneous manifestations of HADDTS. Thus, continued monitoring and follow-up is necessary. Further research on patients with pathogenic variants in *CTBP1* would greatly contribute to the development of diagnostic and management strategies.

**TABLE 1:** Summary of clinical and molecular features of all patients with CTBP1 variants.

	P1 Beck et al. (2016)	P2 Beck et al. (2016)	P3 Beck et al. (2016)	P4 Beck et al. (2016)	P5 Sommerville et al. (2017)	P6 Beck et al. (2019)	P7 Beck et al. (2019)
**Age**	8 y	20 y	9 y	12 y	16 y	22 y	6 y
**Sex**	Male	Male	Female	Female	Female	Female	Male
**Family history**	+	-	-	-	-	-	-
**Growth retardation**	+	+	+	+	+	+	+
**Low Weight (kg)**	**Birth**	**Birth**	**Birth**	**Birth**	NA	NA	NA
	Wt:24%	Wt:16%	Wt: 58%	Wt:95%			
	Ht:86%	Ht:73%	Ht:31%	Ht:81.6%			
	**Current**	**Current**	**Current**	**Current**			
	Wt:4%	Wt:<1%	Wt:<1%	Wt:5%			
	Ht:7%	Ht:<1%	Ht: 13%	Ht:19%			
**Developmental regression**	-	-	-	-	+	+	-
**Dental defect**	+	+	+	+	NA	+	+
**Ataxia**	+	+	+	+	-	-	+
**Intellectual disability**	+	+	-	+	+ (Serve)	+	+
**Language retardation**	+	+	+	+	+	+	+
**Muscular hypotonia**	+	+	+	+	+	+	+
**Feeding difficulties**	+	+	-	+	+	NA	NA
**Abnormal brain MRI**	+	+	+	-	+	+	-
**facial features**	-	Frontal bossing; deep set eyes	Retrognathia highly arched palate	-	NA	Microcephaly	NA
**Others**	NA	NA	NA	NA	Sunken eyes; Thin tapering fingers; Spinal scoliosis; Contractures of the wrists and elbows	Spinal scoliosis; Contractures of the wrists and elbows	NA
**Variant types**	Missense	Missense	Missense	Missense	Missense	Missense	Missense
**Variants**	c.1024 C>T (p.Arg342Trp)	c.1024 C>T (p.Arg342Trp)	c.1024 C>T (p.Arg342Trp)	c.1024 C>T (p.Arg342Trp)	c.991 C>T (p.Arg331Trp) (NM_001012614.2); i.e., c.1024C>T (p.Arg342Trp)	c.1024 C>T (p.Arg342Trp)	c.1024 C>T (p.Arg342Trp)
**Genetic origin**	Maternal mosaicism	*De novo*	*De novo*	*De novo*	*De novo*	*De novo*	*De novo*

P8 Beck et al. (2019)	P9 Beck et al. (2019)	P10 Beck et al. (2019)	P11 Beck et al. (2019)	P12 Bhatia et al. (2020)	P13 Kadhim et al. (2023)	P14 Jafari Khamirani et al. (2021)	Our study
6 y	10 y	5 y	11 y	8 y	15 y	26 y	3 y
Male	Male	Male	Male	Male	Male	Male	Female
-	-	-	-	-	-	-	-
+	+	+	+	+	+	-	+
NA	NA	NA	NA	**Birth**	NA	-	**Birth**
				Wt:17%			Wt: 3%
				Ht:16%			Ht: 3%
				**Current**			**Current**
				Wt:<0.1%			Wt: 6.7%
				Ht:30%			Ht: 5.7%
-	+	+	-	-	+	-	+
+	-	NA	+	+	+	+	-
-	+	+	+	+	+	-	-
+	+	+	+	+ (Mild)	+	+ (Serve)	+ (Moderate)
+	+	+	+	+	+	+	+
+	+	+	+	+	+	-	-
NA	NA	NA	NA	+	NA	+	+
+	+	+	+	+	+	NA	-
NA		Long face	NA	Long face	NA	-	Microcephaly
							Synophrys
							Low-set ears
							Protruding ears
							Visible beard
NA	NA	Scoliosis; Elbow and keen contracture	NA	Scoliosis; Elbow and keen contracture	CK elevated (slightly); Scoliosis; Claw-feet and hammertoes	Seizure; Nystagmus	Single transverse palmar crease
							Short fifth-finger
							Dislocated radial head
							Pectus excavatum
							Myopia
							Spinal scoliosis
							Mild tricuspid valve regurgitation
Missense	Missense	Missense	Missense	Missense	Missense	Frameshift	Missense
c.1024 C>T (p.Arg342Trp)	c.1024 C>T (p.Arg342Trp)	c.1024 C>T (p.Arg342Trp)	c.1024 C>T (p.Arg342Trp)	c.1024 C>T (p.Arg342Trp)	c.1024 C>T (p.Arg342Trp)	c.1315_1316 del CA (p.Gln439ValfsTer84)	c.371 C>T (p.Ser124Phe)
*De novo*	*De novo*	*De novo*	*De novo*	*De novo*	*De novo*	*De novo*	*De novo*

In this study, a novel pathogenic variant was identified in the *CTBP1* gene of a Chinese patient. The patient exhibited distinctive facial features, growth delay, delayed speech and language development, intellectual disability, and skeletal anomalies. The variant described in the study has expanded the phenotypic spectrum among patients from diverse ethnic backgrounds. While the mechanisms underlying these differences remain unclear, they contribute to the exploration of the biological mechanisms involved in *CTBP1* pathogenesis.

## Data Availability

The original contributions presented in the study are publicly available. The data are deposited in the NCBI Sequence Read Archive repository, accession number PRJNA1019228 (https://www.ncbi.nlm.nih.gov/sra/ PRJNA1019228).
